# Multiplexed CRISPR-Cas system targeting ASFV genes *in vivo*: solution lies within

**DOI:** 10.1128/spectrum.00714-24

**Published:** 2024-08-07

**Authors:** Mengjia Zhang, Yifei Lang, Wentao Li

**Affiliations:** 1 National Key Laboratory of Agricultural Microbiology, Hubei Hongshan Laboratory, College of Veterinary Medicine, Huazhong Agricultural University, Wuhan, China; 2 College of Veterinary Medicine, Sichuan Agricultural University, Chengdu, China; Oklahoma State University College of Veterinary Medicine, Stillwater, Oklahoma, USA

**Keywords:** African swine fever virus, genome editing, genetically engineered pigs, disease-resistant pigs

## Abstract

The emergence and spread of the African swine fever virus (ASFV) posed a significant threat to the global swine breeding industry, calling for innovative approaches benefiting viral containment and control. A recent study (Z. Zheng, L. Xu, H. Dou, Y. Zhou, X., et al., Microbiol Spectr 12: e02164-23, 2024, https://doi.org/10.1128/spectrum.02164-23) established a multiplexed CRISPR-Cas system targeting the genome of ASFV and tested the consequent antiviral activity both *in vitro* and *in vivo*. Application of this system showed a significant reduction of viral replication *in vitro*, while the germline-edited pigs expressing this system exhibited normal growth with continuous guide RNA expression. Although no survival advantage was observed upon ASFV challenge compared with nonengineered pigs, this marks the first attempt of germline editing to pursue ASFV resistance and paves the way for future disease-resistant animal breeding approaches utilizing CRISPR-Cas technology.

## COMMENTARY

African swine fever (ASF) is a highly contagious and deadly viral disease affecting both domestic and wild pigs. ASF is now confirmed to have an expanding distribution across at least 80 countries, in which resides more than 80% of the world’s total swine population. Despite the century-long prevalence history of ASF, the current situation pressingly demands effective prevention and control strategies, namely, the development of efficient vaccines and effective antiviral drugs. The complexity of the African Swine Fever Virus (ASFV), the causative agent of ASF, is proven to be the major obstacle affecting the development of antiviral intervention strategies. ASFV particles have a complex 5-layer structure and a large DNA genome encoding more than 160 proteins, most of which still lack proper elucidation of their biological properties and functions (1, 2). Among the proteins that were characterized, researchers identified and confirmed large numbers of functional overlaps between them. The existence of such mechanisms could directly lead to robust immune evasion or immune suppression capabilities of the virus, which hinders viral protein-targeting antiviral interventions (3–6). In addition, the ASFV also exploits multiple complex infection mechanisms such as hijacking the cellular efferocytosis pathway and makes use of the generated apoptotic bodies for efficient cell-to-cell transmission (7), which have almost shut the door on the development of vaccine candidates following the traditional methodologies.

The fight against the ASFV demands immediate application of innovative antiviral approaches, especially those *de novo* techniques which were initially considered irrelevant, complicated, or interdisciplinary. To this end, Zheng et al. set out to explore the potential of multiplexed CRISPR-Cas systems against ASFV infection (8). The viral genome of the ASFV is a linear DNA molecule that replicates within the host cell during viral infection, making it vulnerable to guide RNA-mediated CRISPR-Cas9 hydrolysis, which could also become more efficient if the system is already equipped by the host. The researchers first designed a multiplex CRISPR-Cas system targeting several loci within the ASFV genome, which shall fragment the viral DNA and render it nonfunctional. The designed system was then integrated into the genome of COS-7 cells, while infection experiments indicated that ASFV replication was significantly reduced in cell clones with higher levels of Cas9 expression. Meanwhile, based on the *in vitro* findings, the authors integrated the same system into pig cells and henceforth generated transgenic pigs. The transgenic animals had optimal growth properties and could maintain Cas9 expression throughout the experiment. Unfortunately, the multiplex CRISPR-Cas system did not maintain noticeable antiviral effects *in vivo*, as those transgenic pigs did not show enhanced resistance to ASFV infection in comparison with the wild-type pigs during the virus challenge experiment. Differing from *in vitro* infection models, the integrated system under *in vivo* conditions has complicated and often unknown obstacles, such as the fully functional immune system and other host factors. Various possibilities that cause incompetency of the Cas9 enzyme might occur, leading to partial or complete incapacitation. Nevertheless, as a valuable first attempt, this study managed to successfully express a complex ASFV-resistant CRISPR-Cas system in pigs and shed light on future researches with alternative settings of the same approach.

Currently, with the rapid development and innovation of gene-editing technology, disease-resistant breeding has progressively become an important resource to prevent and control viral diseases, which can limit the occurrence of diseases by inhibition of viruses from initiating a positive infection. Genetically modified organism (GMO) plants, such as soybeans and corns with various disease-resistant characteristics, have been widely used in the food industry. Less than a decade ago, Whitworth et al. used the CRISPR-Cas9 method to generate pigs lacking functional CD163, the receptor of Porcine Reproductive and Respiratory Syndrome Virus (PRRSV), and the gene-edited pigs showed no fever or respiratory signs, lung pathology, viremia, or antibody response and remained healthy post virus challenge (9). Building upon this study, Xu et al. have produced CD163 and pAPN double-knockout pigs, which showed resistance to PRRSV and Transmissible Gastroenteritis Virus, while having limited susceptibility to porcine deltacoronavirus, an emerging zoonotic coronaviral pathogen discovered in 2012 (10). Researchers have also used gene-editing technology to produce the first calf resistant to bovine viral diarrhea virus (11), a major viral disease that costs the U.S. cattle sector billions of dollars each year. The first gene-edited swine breed for commercial usage with PRRS-resistant characteristics is also on the way to mass production (12). Could there be an ASFV-resistant swine breed in the future? Zheng et al. make a first valuable attempt and inspired a vast possibility for this aspect. There will still be challenges, including insurance of the safety and efficacy of GMO animals with thorough ethical considerations. The ongoing research in this field holds promise for developing sustainable strategies to enhance animal health and welfare while preventing the spread of infectious diseases, especially those high contagious and lethal viral diseases currently posing threats to the field.
